# A Unique Case of Extramedullary Relapse in Acute Lymphoblastic Leukemia: Testicular to Ocular, Cardiac, and Colonic Involvement and the Role of Sperm Phenotyping in Diagnosis—Case Report and Literature Review

**DOI:** 10.3390/jcm14020405

**Published:** 2025-01-10

**Authors:** Alina Camelia Cătană, Maria-Gabriela Vlădoiu, Mariana Sandu, Ariela Olteanu, Liliana Mocanu, Elena Mihai, Minodora Teodoru, Claudiu Matei, Renata Zahu, Zsofia Varady, Lidia Mondoc, Cristina Noor, Andreea Moicean, Geanina Mera

**Affiliations:** 1 County Clinical Emergency Hospital Sibiu, 550245 Sibiu, Romania; csandu12@yahoo.com (M.S.); alolteanu@yahoo.co.uk (A.O.); lilianacmocanu@yahoo.com (L.M.); elenami19@yahoo.com (E.M.); minodora.teodoru@ulbsibiu.ro (M.T.); lidia2jc@yahoo.com (L.M.); cristinalazea@yahoo.com (C.N.); geanina.mera@gmail.com (G.M.); 2 Faculty of Medicine Sibiu, Lucian Blaga University of Sibiu, 550169 Sibiu, Romania; claudiumatei@yahoo.com; 3 Medlife Polisano Hospital, 550172 Sibiu, Romania; 4 Amethyst Radiotherapy Center, 407280 Cluj Napoca, Romania; zahurenata@yahoo.com; 5 Fundeni Clinical Insitute, 022328 Bucuresti, Romania; varadyzsofia@gmail.com; 6 Södersjukhus Hospital, 11883 Stockholm, Sweden; andreea.moicean@gmail.com

**Keywords:** acute lymphoblastic leukemia T, testicular relapse, orbital, cardiac, spermatic fluid immunophenotyping

## Abstract

Acute lymphoblastic leukemia (ALL) is a malignant condition of lymphoid progenitor cells that primarily affects the pediatric population, but also adults. The 5-year survival rate is 90% in children and approximately 40% in adults, with survival increasing through the use of peripheral stem cell allotransplantation (SCT). The relapse rate after stem cell transplantation (SCT) in adult acute lymphoblastic leukemia (ALL) patients ranges from 35% to 45%, making relapse a major cause of death in this population. **Background:** We present an atypical case of late testicular involvement in ALL in a 50-year-old man diagnosed with ALL pro-T in remission post-chemotherapy (GMALL 2003 protocol) and allogeneic stem cell transplantation (alloSCT) from a related donor. **Methods:** This case describes a 50-year-old male with ALL pro-T who experienced three rare extramedullary relapses post-chemotherapy and alloSCT. Five years after remission, he had a unilateral testicular relapse confirmed by immunophenotyping of spermatic fluid. **Results:** Despite no bone marrow involvement, he was treated with chemotherapy, intrathecal therapy, and bilateral testicular radiotherapy. He later relapsed in the orbit, controlled by radiotherapy, followed by a third relapse in the heart and colon. **Conclusions:** This case highlights the unusual sites and consecutive nature of extramedullary relapses in adult ALL.

## 1. Introduction

Acute lymphoblastic leukemia (ALL) is the most common malignancy in children. Extramedullary relapses (EMR) after stem cell transplantation (SCT) have been primarily reported in this patient population [1]. EMR cases in adults with acute lymphoblastic leukemia (ALL) who have undergone allogeneic transplantation are rare, often limited to case reports, and frequently involve comparisons with similar pediatric cases. Relapses of ALL predominantly occur in the bone marrow (BMR), accounting for about 60% of cases, but also extramedullary (EMR) [2]. T-cell acute lymphoblastic leukemia (T-ALL) accounts for 10–15% of ALL cases in adults and 15–25% in children, with a more aggressive clinical course and higher incidence of extramedullary relapses compared to B-cell acute lymphoblastic leukemia (B-ALL), which is more common, making up 75–80% of childhood cases and 25–40% of adult cases [2]. Three-year survival is 8.5% in BMR vs. 30.1% in EMR. More than that EMR occurs later post-transplant compared to BMR at 10 vs. 4 months. Late relapses at 5–10 years post-transplant are rarer [2,3]. EMR is most commonly seen in the CNS and testicles, with isolated testicular relapses occurring in less than 1% of adults, while pediatric populations show rates of 2% to 5% [4]. Relapses have also been reported at the level of the breast, ovary, uterus, cervical region, pancreas, kidney, intestine, heart, eye, lymph nodes, and skin [5]. Rarely described are concurrent extramedullary determinations or the presence of consecutive EMR relapses in different locations, without concurrent or distant medullary relapse. Typically, EMR is confirmed through biopsy or tissue ablation. This report describes a unique case of late testicular relapse in an adult patient with pro-T ALL who underwent allogeneic SCT and later experienced concurrent orbital, cardiac, and colonic relapses [6,7]. The testicular relapse was confirmed through immunophenotyping of the spermatic fluid, a method not previously documented in the literature. This case highlights the complexities of managing late EMR in adults with ALL and emphasizes the need for increased awareness of such rare occurrences [8]. Our literature review did not identify any documented cases of testicular relapse confirmed by morphology and immunophenotyping of spermatic fluid. Additionally, we found no reports of leukemic involvement in the testicle, orbit, heart, or colon. Specifically, the occurrence of leukemia in these four organs—testicle, orbit, heart, and colon—in adults with T-cell ALL and relapses occurring distant from transplantation has not been documented.

## 2. Materials and Methods

We present the case of a 50-year-old patient with a tissue mass in the left anterior mediastinum, first identified during a routine chest X-ray in 2013 and confirmed by tomography. The patient did not seek further evaluation until November 2016, at which time the mass had grown to 13.5 × 14 × 12 cm. A reductional tumorectomy was performed in December 2016. Histopathological examination (HP) and immunohistochemistry (IHC) classified the mass as a mixed thymoma, AB type, predominantly B2 (cortical) with restricted areas (10%) of type A thymoma (medullary). Postoperative TAP CT in January 2017 showed no evidence of tumor recurrence. In June 2017, the patient presented to the hematology department with subfebrile syndrome, herpes labialis, oropharyngeal mucositis, dysphagia, and hepatomegaly. The patient underwent all necessary laboratory tests and paraclinical examinations for establishing a diagnosis, which are presented in Table 1.

## 3. Results

The patient was diagnosed with acute lymphoblastic leukemia (ALL), pro-T subtype, according to the 2016 WHO classification. He received treatment in accordance with the GMALL 2003 protocol, starting with the pre-phase and induction therapy. Complete remission was achieved both morphologically and phenotypically, with minimal residual disease (MRD) testing returning negative results.

Following the second course of treatment, the patient developed Clostridium difficile enterocolitis, which was successfully treated with antibiotic therapy. He also underwent cranial radiotherapy at a dose of 24 Gy, along with triple intrathecal (IT) therapy (methotrexate, cytarabine, and dexamethasone) as prophylaxis against meningoencephalic involvement.

After consolidation therapy, the patient experienced multiple infectious complications, a reactivation of Clostridium difficile, and the detection of a positive hepatitis B surface antigen (AgHBs). These infections were managed with appropriate antibiotic therapy, and entecavir was initiated for the hepatitis B infection, even though the HBV DNA level was low (164 IU/mL) and not accompanied by hepatitis (clinical or laboratory hepatic abnormalities). Entecavir was prescribed, as it is reimbursed and recommended for patients undergoing immunosuppressive therapy.

In February 2018, the patient successfully underwent consolidation therapy with allogeneic peripheral blood stem cell transplantation from a fully HLA-compatible (10/10) related donor, his sister, sharing the same blood group and positive CMV serostatus. A myeloablative conditioning regimen with busulfan and cyclophosphamide (BUCy) was used. Tacrolimus and methotrexate were given for GVHD prophylaxis. He successfully engrafted on day +14, achieving complete donor chimerism.

By day +45, tacrolimus-induced acute kidney injury necessitated switching to sirolimus. On day +45, the patient developed grade III cutaneous GVHD, treated with methylprednisolone (2 mg/kg/day), which resulted in complete resolution of GVHD symptoms. Immunosuppression was discontinued six months post-transplant without GVHD recurrence. The only notable late complication was bilateral cataracts, which were surgically corrected with lens implants.

The patient underwent regular hematological follow-ups, including immunophenotypic evaluation for minimal residual disease in the bone marrow and imaging to monitor for recurrence of the mediastinal tumor.

In February 2022, he contracted a SARS-CoV-2 infection, which was treated symptomatically at home. A Thoraco-abdomino-pelvic CT scan performed the same month was within normal limits.

By March 2022, the patient reported lumbar spine pain. Imaging revealed disc protrusions at the L2–S1 levels, along with moderate spinal canal stenosis at L4–L5.

In May 2022, the patient noted discomfort and enlargement of the left testicle, without associated pain, local signs of inflammation, fever, chills, or scrotal trauma. The right testicle appeared normal on examination. He consulted the urology department, where primary testicular carcinoma was suspected. However, the patient delayed surgical intervention for one month.

In June 2022, he presented to the hematology department with a large, 8 cm, left testicular tumor, which was hard, nodular, and without adjacent clinical signs.

**Figure 1:**
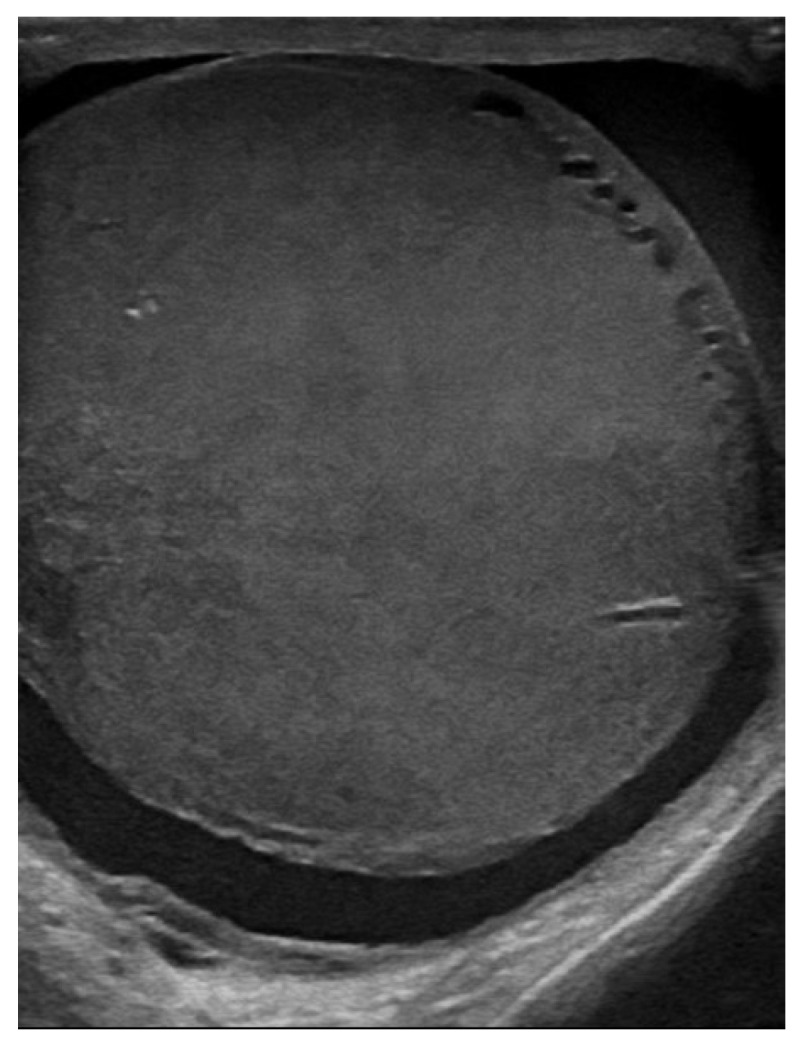
Axial ultrasound section of the left testicle: increased size, inhomogeneous echostructure, and alternating hypoechoic areas.

**Figure 2:**
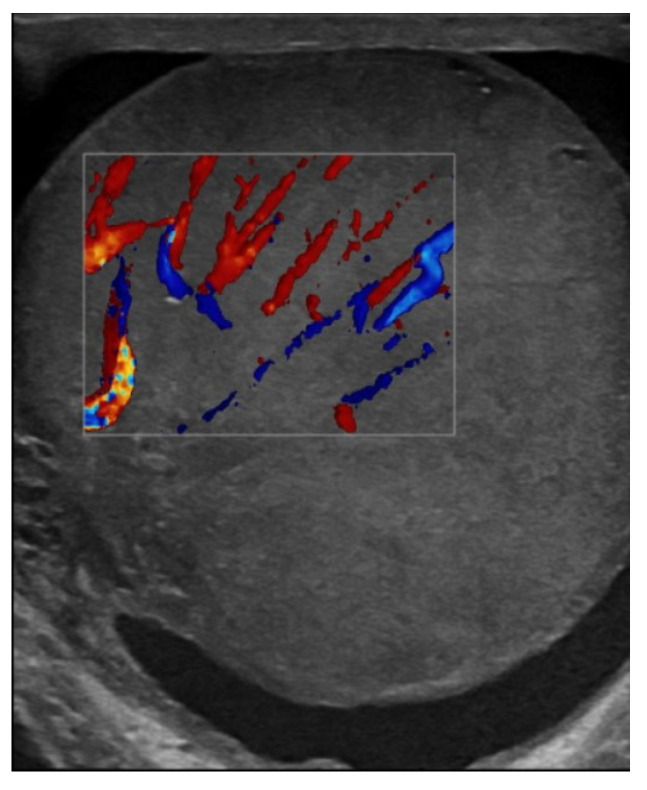
Vascular signal present in Doppler.

**Figure 3:**
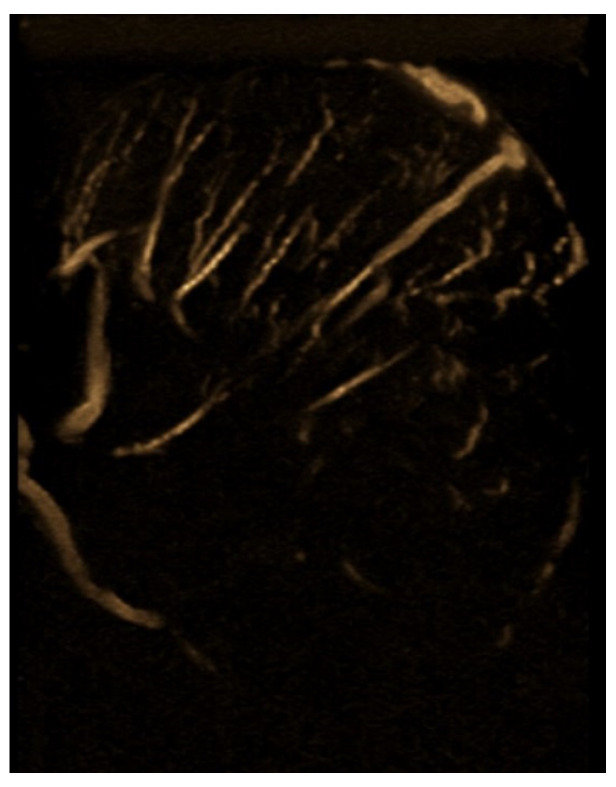
Vascular signal present in B-Flow.

Testicular ultrasound revealed an enlargement of the left testicle approx. 63/55 mm, with diffuse heterogeneous infiltrated structure and vascular signal present on Doppler and B-Flow imaging, without specific intraparenchymal masses detectable ultrasonographically, with a transonic vaginal reaction with a diameter of approximately 7 mm (Figure 1, Figure 2 and Figure 3).

The hematologist, in collaboration with the radiologist, raised the suspicion of testicular relapse, given the patient’s history of acute lymphoblastic leukemia (ALL). However, it was noted that prior treatments, including radiotherapy, chemotherapy, and immunosuppressive therapy for graft-versus-host disease, may have been risk factors for the development of a primary testicular neoplasm.

**Figure 4:**
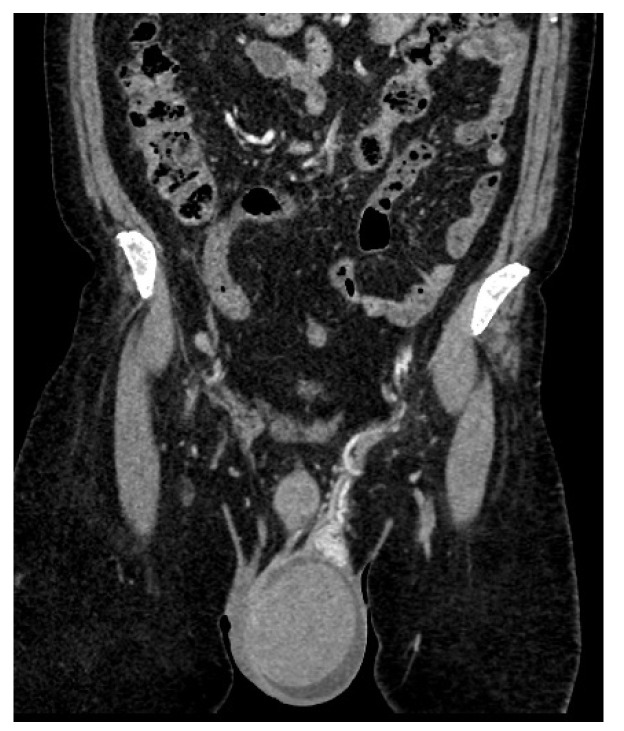
Abdomino-pelvic CT with contrast substance, coronal plane sections: Left testicle.

**Figure 5:**
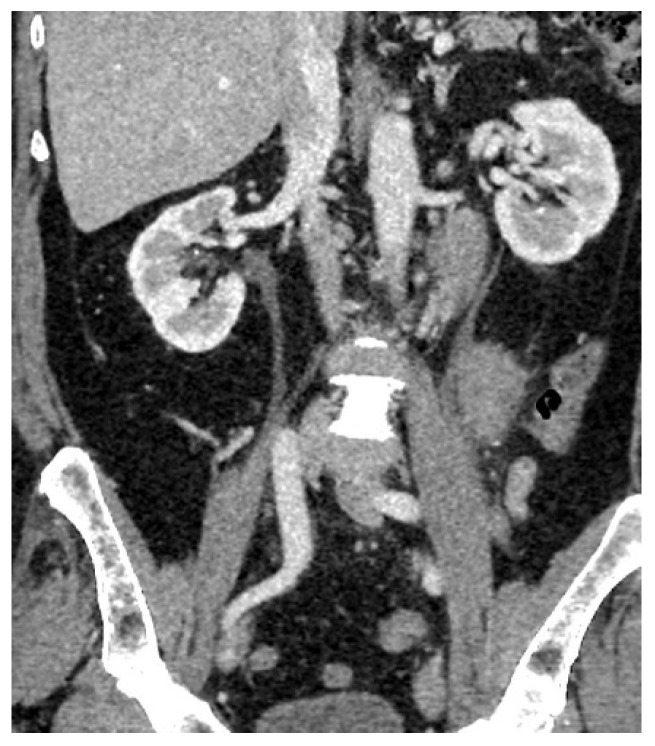
Abdomino-pelvic CT with contrast substance, coronal plane sections: Adenopathies along the path of the left testicular vein.

A thoraco-abdomino-pelvic CT scan revealed an enlarged left testicle measuring approximately 100 × 60 × 60 mm, with increased native densities and prominent peripheral vascularity. The testicle exhibited discreet iodophilia, with an adjacent fluid lamina and contrast uptake in the epididymis, spermatic cord, and left hemiscrotum. Additionally, tissue nodules with heterogeneous iodophilia were observed along the course of the left testicular vein, extending to the left renal hilum. Sub-hilar adenopathies were noted, measuring 42 × 26 × 36 mm, 31 × 27 × 54 mm, and 13 mm, respectively. Incidental findings included gallbladder lithiasis and bilateral renal microlithiasis (Figure 4 and Figure 5).

The possibility of testicular relapse was discussed, and the method for establishing a definitive diagnosis was debated. Testicular ablation would have been both diagnostic and therapeutic, but it would have delayed chemotherapy. Additionally, considering the presence of adenopathies along the path of the left testicular vein, it would have increased the risk of systemic disease recurrence. Testicular biopsy was contraindicated due to the rich vascularization of the testicle and its tension.

Medullary relapse was excluded morphologically, immunophenotypically, and immunohistochemically—no atypical cells were identified.

Biological investigations demonstrated the following: white blood cell count (WBC) of 7460/mm^3^, hemoglobin (Hb) of 15.3 g/dL, hematocrit (Ht) of 43.1%, platelet count (Pl) of 237,000/mm^3^, lactate dehydrogenase (LDH) of 190 U/L, and beta-human chorionic gonadotropin (β-HCG) < 0.10, which is within the normal range and typically elevated in primary testicular tumors.

**Figure 6:**
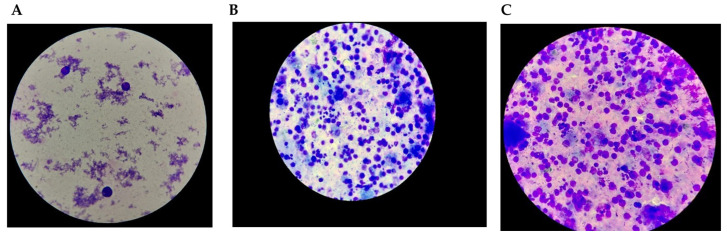
Spermiogram and cytospin. (**A**) Spermiogram—simple smear technique by spreading; (**B**) Cytospin—concentration technique—mononuclear, presence of microbial flora; (**C**) Spermiogram—mononuclear cells with blast-like morphology.

A spermogram conducted in July 2022 showed the following results: appearance and color were whitish-yellow and slightly opalescent; viscosity was normal at 2 h; volume was 3.0 mL (reference range: 2–6 mL); pH was 8.0 (reference range: 7.2–8.0). The sperm count was 0.0 million sperm/mL (reference range: 20–200 million sperm/mL), and round cells were noted at 5.5 million/mL (Figure 6 A–C).

Morphological analysis, performed on smears (by spreading and cytospin) stained with May–Grünwald–Giemsa (MGG), revealed cellularity composed of mononuclear cells with morphology suggestive of blast cells (lymphoblasts), macrophages, neutrophil polymorphonuclear cells, and very rare germ cells. Additionally, microbial flora was present in the specimen.

Given the presence of mononuclear cells with a blast-like appearance, flow cytometry was recommended to further characterize these cells and confirm their identity.

Therefore, as an alternative to orchiectomy for establishing a definitive diagnosis, the discussion and implementation of immunophenotyping of the seminal fluid were considered. This method, to our knowledge, has not been performed in Romania, and, consequently, there is no standardized protocol for it. Additionally, we did not find any relevant data in the literature regarding this approach.

**Figure 7:**
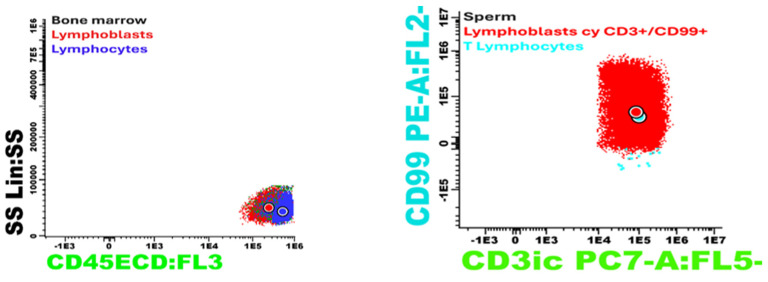

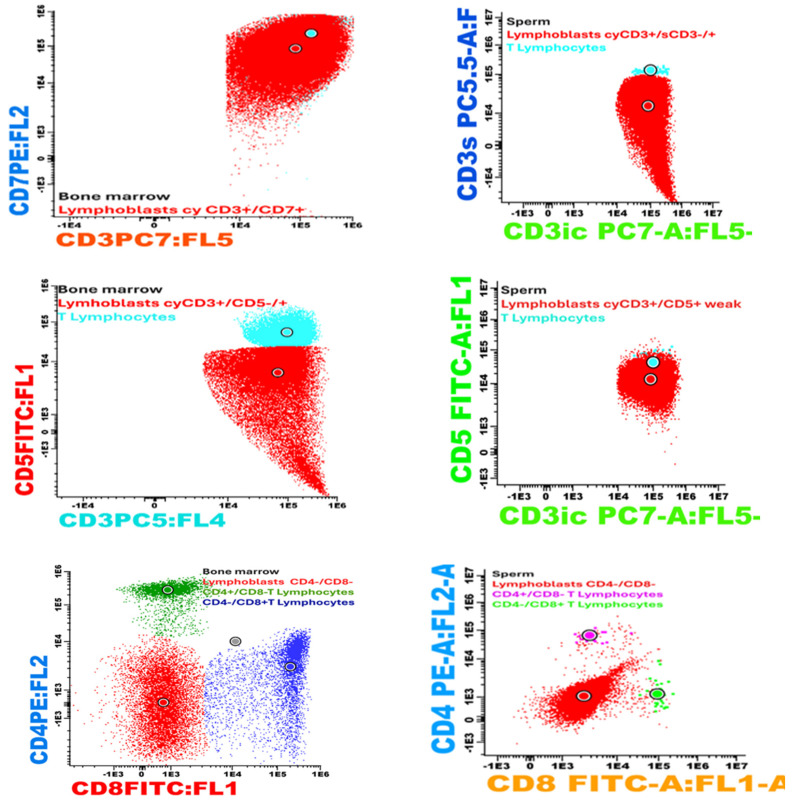
Immunophenotyping of the seminal fluid using a panel including CD45, CDCD3s, CD3ic, CD4, CD5, CD8, and CD99.

Immunophenotyping of the seminal fluid using a panel including CD45, CDCD3s, CD3ic, CD4, CD5, CD8, and CD99 identified 36% atypical cells with the following immunophenotype: CD3ic+, CD3s−/+, CD99+, CD4−, and CD8−. This result indicated that the pathological product is infiltrated with atypical cells with an immunophenotype consistent with the underlying disease (T-ALL). This analysis was performed using the Dx FLEX 3 laser and 13-color analyzer (utilizing five colors) (Figure 7).

Once the diagnosis of testicular relapse of T-cell acute lymphoblastic leukemia (pro-T ALL) was confirmed, the optimal therapeutic option was discussed. Considering the limited data in the literature, primarily focused on children, as well as the adenopathies along the path of the left testicular vein, a chemotherapy regimen of HyperCVAD Block A was chosen.

Additionally, four intrathecal administrations of triple therapy were administered—dexamethasone, cytarabine, and methotrexate, along with treatment with entecavir one tablet/day orally (0.5 mg).

The assessment conducted after the aplasia confirmed the absence of medullary relapse in October 2022.

Testicular ultrasound revealed the left testicle had dimensions of 52/28 mm, with a heterogeneous echotexture due to the presence of scattered microcalcifications and mild dilatation of the testicular veins, with minimal adjacent edema, and the presence of vascular signal. Moderate dilatation of the pampiniform plexus was observed. Peritesticular fluid collection in a moderate quantity was noted. Bilateral epididymis with microcalcifications was observed. The inguinal canals were free.

**Figure 8:**
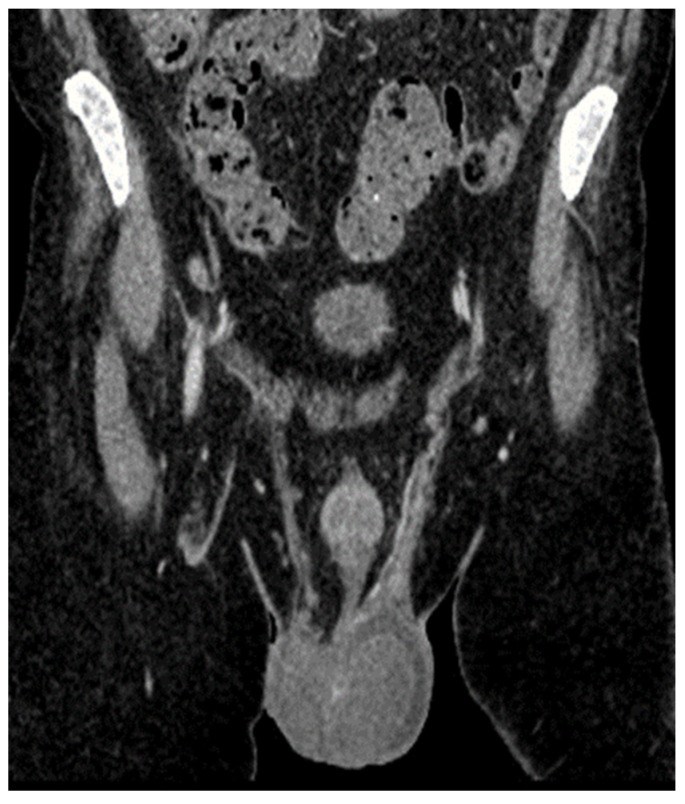
Comparative abdomino-pelvic CT examination—dimensionally reduced left testicle.

CT examination showed regressive dimensional changes in the left testicle, with current dimensions of 50/40/40 mm compared to 100/60/60 mm previously, with reduced iodophilia compared to the previous examination (Figure 6). Adenopathies along the path of the left testicular vein appeared reduced in size, with a maximum diameter of up to 20 mm (Figure 8).

From a hematological perspective, partial remission was achieved, with an 80% reduction in the size of the testicle and a 50% reduction in abdominal masses. At this stage, the treatment options included either testicular ablation or a combination of testicular and abdominal radiotherapy, potentially followed by testicular ablation. After careful consideration, the decision was made to proceed with testicular and abdominal radiotherapy targeting the adenopathies along the left testicular vein.

Radiotherapy was administered to the scrotal sac, both testicles, and the abdomino-pelvic adenopathies, delivering a total dose of 24 Gy over 12 sessions from 5 September to 20 September 2022.

On November 1, 2022, a CT scan of the abdomen and testicles demonstrated regressive dimensional changes in the left testicle, which measured 4.5 × 4 × 3 cm, compared to its previous size of 5 × 4 × 4 cm. The testicle exhibited non-iodophilic characteristics, with fluid and parafollicular densities but no detectable post-contrast iodophilia. The left spermatic cord appeared thickened and moderately iodophilic. Additionally, the adjacent adenopathies in the cranial portion of the left testicular vein showed a reduction in size, with a maximum diameter of 1.5 cm.

A subsequent PET/CT re-evaluation on 24 November 2022, revealed no detectable FDG-hypercaptant lesions suggestive of malignancy. A hematological re-evaluation in December 2022, including bone marrow aspiration and immunofluorescence, confirmed minimal residual disease (MRD) to be negative.

On 17 January 2023, the patient was hospitalized due to the onset of left orbital exophthalmos, which had begun two weeks prior. Clinical examination revealed left eyelid ptosis and left globe protrusion, with asymmetric pupils and a tumor mass in the superoexternal angle, while the right eye showed no clinical changes. The visual field examination indicated an enlargement of the blind spot in the left eye, while the right eye remained unchanged.

Biological parameters at that time included a white blood cell count of 6030/mm^3^, hemoglobin of 13 g/dL, hematocrit of 38.1%, and platelets at 217,000/mm^3^, with no blasts detected in the peripheral blood. Bone marrow aspiration and immunophenotyping again confirmed MRD to be negative.

A CT scan of the thoraco-abdomino-pelvic region performed on 20 January 2023, revealed no significant changes.

**Figure 9:**
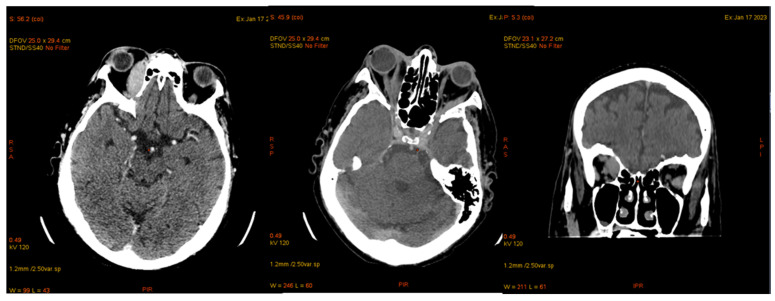
Cerebral CT—tumoral mass right and left eye.

Cranial MDCT examination on 7 January 2023, showed grade II/III right exophthalmos and pseudophakia in the right eye. An intraorbital, extraconal tissue mass was noted on the superointernal wall of the right orbit, measuring approximately 1.7 × 4.2 cm and displacing the medial portion of the right internal rectus muscle while contacting the middle portion of the right optic nerve. There was also a small area of discontinuity in the right lesser wing of the sphenoid bone, characterized as osteolytic, with an extraneural tissue component measuring approximately 0.7 × 1.2 cm located at the anterior floor of the middle cranial fossa. Grade II left exophthalmos was observed, with no changes in the left eye or retro-ocular fat. Minimal thickening of the left internal rectus muscle was noted in the anterior half, with a maximum diameter of approximately 5 mm, and the left lateral rectus muscle showed a posterior half-thickness of approximately 6 mm (Figure 9).

**Figure 10:**
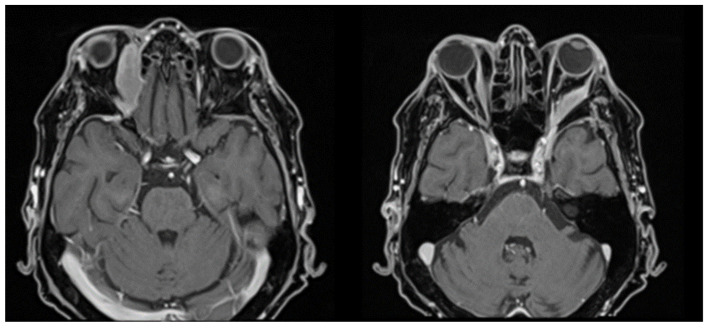
Cerebral MRI—tumoral mass LE.

Cerebral MRI conducted on January 30th revealed a tissue mass in the right intraorbital, extraconal region, measuring approximately 1.8 × 1.8 × 4 cm (length × width × height). This mass exhibited intense restriction and gadolinium enhancement characteristics. It impressed upon and deviated the right external rectus muscle, while making minimal contact with the right optic nerve. Additionally, the right external rectus muscle showed diffuse hypertrophy in the posterior two-thirds, with intense restriction and gadolinium enhancement, measuring approximately 1.2 cm in maximum transverse diameter (Figure 10).

A wide incision biopsy was performed on the orbital tumor, with a significant incision made along the left eyebrow arch. Histopathological examination (HP) and immunohistochemistry (IHC) revealed two fragments, each measuring 0.7 cm, containing tissue invaded by CD3- and CD5-positive lymphoblasts, confirming the presence of T-cell lymphocytes. Rare CD20-positive B lymphocytes were also noted, while CD23 was negative. Additionally, CD10 was positive in the stroma but negative in the lymphocytes. The biopsy results indicated positive terminal deoxynucleotidyl transferase (TdT) and a Ki-67 proliferation index of 90%. The 7 mm friable sample did not allow for additional immunofluorescence (IF) marker analysis.

**Figure 11:**
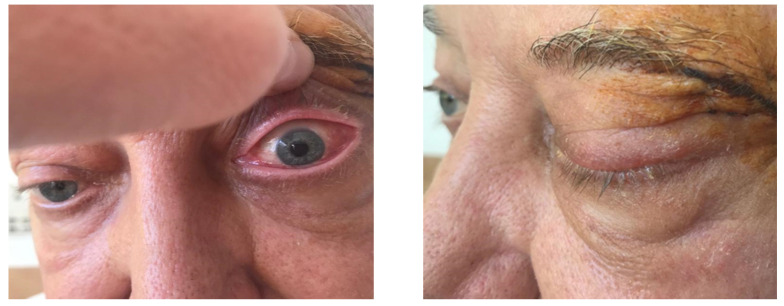
Left eye tumor, eyelid ptosis, exophthalmos.

An ophthalmologic examination conducted on 15 February 2023, showed best-corrected visual acuity of 0.8 for the right eye (OD) and perception of light for the left eye (OS). Intraocular pressure measurements were 16 mmHg for OD and 12 mmHg for OS. Exophthalmometry readings were 22 mm for OD and 24 mm for OS. The diagnosis included ischemic optic neuropathy (noted on 8 February 2023), anterior ischemic optic neuropathy, left eye exophthalmos, left eye ptosis, and tumor formation in the orbit. Cerebrospinal fluid analysis showed no evidence of blast cells (Figure 11).

The ophthalmologic evaluation demonstrated a visual acuity of 20/20 in the right eye without correction, while the left eye was limited to the perception of light. Intraocular pressures were recorded at 13 mmHg for the right eye and 12 mmHg for the left eye. Exophthalmometry revealed right eye protrusion of 22 mm and left eye protrusion of 26 mm. Ocular motility was intact in the right eye, whereas the left eye exhibited global immobility and inferior chemosis.

**Figure 12:**
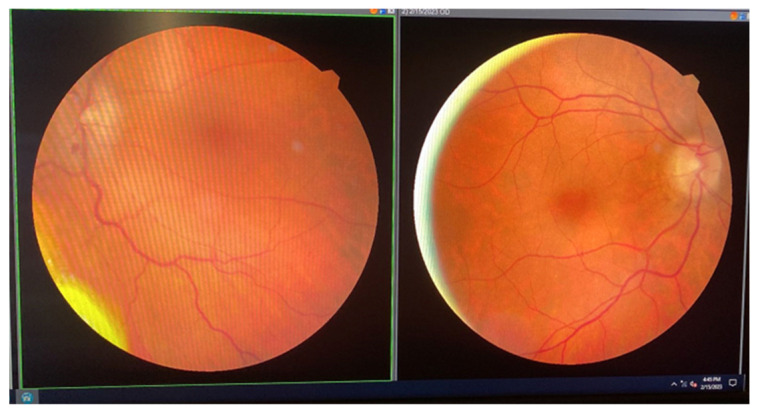
Ocular fundus.

Fundoscopic examination showed an intact disc margin in the right eye, accompanied by slight fading and a non-absorbed hemorrhage at the 6 o’clock position. The left eye exhibited a faded disc margin with discoloration and a similar hemorrhage that had not absorbed. Visual field testing for the right eye indicated enlargement of the blind spot and decreased retinal sensitivity in the central area. Optical coherence tomography (OCT) revealed no significant changes in the right eye, while findings in the left eye indicated optic nerve atrophy consistent with non-arteritic ischemic optic neuropathy (NAION) and the presence of an orbital tumor (Figure 12).

On 14 February 2023, the patient was administered a regimen of high-dose methotrexate, asparaginase, and calcium folinate, which was well tolerated and resulted in regression of ptosis and a discrete improvement in exophthalmos. However, the patient developed an infection with Clostridium difficile, which was effectively treated with intravenous Tygacil and oral vancomycin.

Histopathology (HP) and immunohistochemistry (IHC) results from the ocular biopsy were received on 16 February 2023. Given the diagnosis of a second relapse, a decision was made to initiate a chemotherapy regimen consisting of nelarabine—a previously unused cytostatic agent—combined with etoposide and cyclophosphamide.

**Figure 13:**
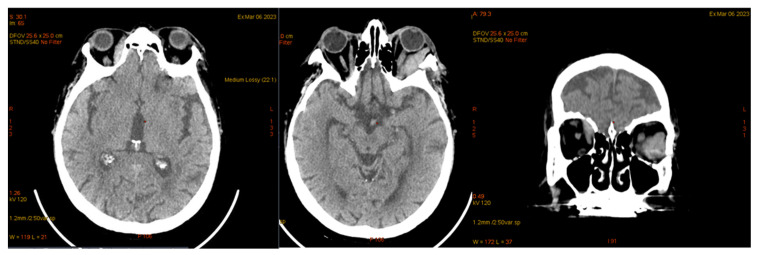
Cerebral computed tomography—same characteristics as the previous one.

On 6 March 2023, the patient was diagnosed with influenza B, which resulted in a delay in the planned chemotherapy regimen. A subsequent CT scan of the brain and thoraco-abdominal region was performed to assess the patient’s condition (Figure 13).

In March 2023, imaging studies revealed an intraorbital, extraconal mass on the right side that was adherent to the supero-internal wall of the orbit. The mass showed a reduction in size, now measuring approximately 0.9 × 2 cm, compared to the previous dimensions of 1.7 × 4.2 cm. Notably, there was no mass effect on the right medial rectus muscle and a reduced contact area with the optic nerve. In contrast, the left side exhibited grade II exophthalmos, with significant thickening of the left medial rectus muscle, measuring approximately 1.4 × 4.4 × 2.5 cm (length × anteroposterior × cross-section), indicating a tissue mass occupying the posterior half of the orbit, currently in contact with the optic nerve.

**Figure 14:**
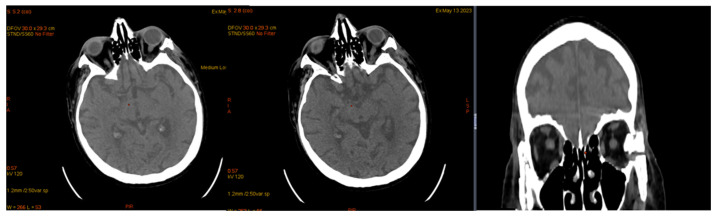
Cerebral CT, May 2023. The bilateral intraorbital tissue masses are no longer evident compared to previous examinations, and there are no areas of ischemia or meningoencephalic infiltration.

A CT scan (Figure 14) of the thoraco-abdomino-pelvic region conducted in March 2023 revealed sub-centimeter-sized adenopathies along the left testicular vein, as well as minimal diffuse thickening of the cecal and ascending colon walls, accompanied by adjacent pericolonic fat stranding. There was moderate distension with mixed content at the cecum-ascending colon level, suggestive of post-enterocolitis due to Clostridium.

From 13 March to 31 March 2023, the patient underwent radiation therapy, receiving a total dose of 30 Gy delivered in 15 fractions to the bilateral orbit, employing the SIB-IMRT/VMAT technique, which resulted in the remission of the orbital tumors.

On 12 April 2023, the patient presented with a respiratory viral infection characterized by symptoms of bronchitis and acute sinusitis, leading to a delay in the chemotherapy regimen.

By 2 May 2023, the patient exhibited an altered general condition with pronounced asthenoadynamic syndrome, bradycardia (30 beats per minute), normal blood pressure, and left monobrachial paresis. Bowel transit disorders were noted, alternating between diarrhea and constipation. The biological findings revealed no blasts detected in the peripheral blood or bone marrow, and the patient tested negative for Clostridium difficile.

**Figure 15:**
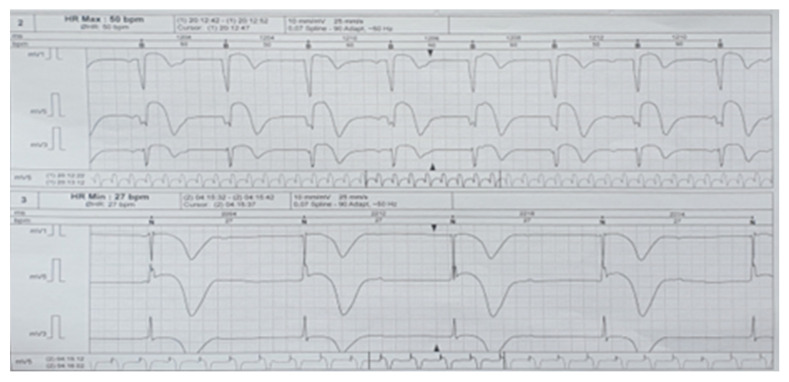
Complete atrioventricular block.

The 24 h Holter ECG examination recorded complete atrioventricular block (BAV) with junctional and ventricular escape rhythm with QRS complexes of two different morphologies, with a maximum heart rate (AV max) of 73 bpm, a minimum heart rate (AV min) of 27 bpm, and a mean heart rate (AV average) of 37 bpm (Figure 15).

**Figure 16:**
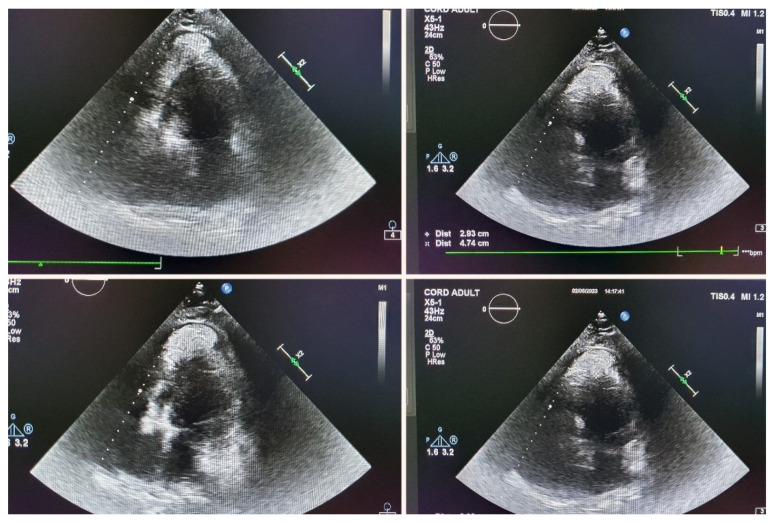
Echocardiographic evaluation.

Echocardiographic evaluation revealed a dilated left atrium with a thickened lateral wall. The left ventricle was of normal size, but the interventricular septum was thickened and demonstrated global hypokinesis, resulting in slightly impaired systolic function, with an ejection fraction of 45%. Additionally, an oval-shaped pericardial formation was identified at the apex of the left ventricle, measuring 30 × 48 mm. This mass was in contact with the left ventricular wall and appeared to invade the myocardium. A fine pericardial layer was noted lateral to the left ventricular wall, with no significant valvular pathology observed (Figure 16).

The evaluation of cardiac markers revealed their reaction, suggestive of myocardial injury and heart failure, as follows: CKMB: 7.6 mg/mL; Myoglobin: 207 mg/mL; Troponin: 1.64 mg/dL; BNP: 2490 pg/mL; and D-dimer: 3300 mg/mL.

Considering the clinical presentation and paraclinical findings, cardiac involvement was interpreted as resulting from atrial and ventricular myocardial infiltration, which led to conduction disturbances and the development of heart failure characterized by a slightly reduced left ventricular ejection fraction. In response to these findings, a comprehensive treatment regimen was initiated, including antiplatelet therapy, a statin, a loop diuretic, an angiotensin receptor–neprilysin inhibitor (ARNI), an SGLT2 inhibitor, and a mineralocorticoid receptor antagonist. The specific medications prescribed were aspirin (75 mg/day), atorvastatin (40 mg/day), sacubitril/valsartan (24/26 mg twice daily), dapagliflozin (10 mg/day), and furosemide/spironolactone (20/50 mg/day). This multi-faceted approach aims to manage heart failure symptoms, improve cardiac function, and mitigate further cardiac complications.

**Figure 17:**
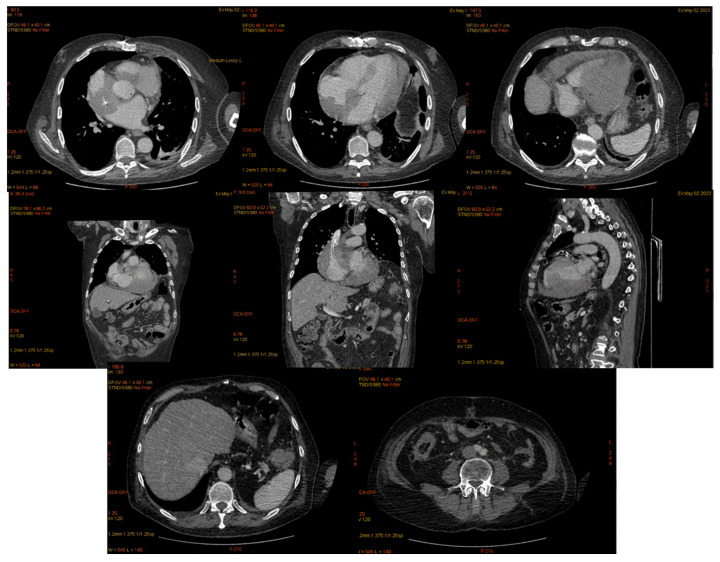
Cardiac computed tomography.

The cardiac computed tomography performed in May 2023 revealed parietal thickening of the heart with iodophilic areas within the cavities. The right atrium (RA) displayed uneven thickening of the supero-lateral-posterior wall, measuring approximately 3 cm, resulting in an irregular contour of the RA and left atrium (LA) lumens. The right ventricle (RV) showed thickening of the postero-superior wall at about 1.5 cm, while the left ventricle (LV) had uneven thickening of the infero-lateral-medial wall, measuring 2.5 cm. Additionally, the cecum was dilated with thickened walls (approximately 1.1 cm), and the descending colon exhibited anterior-wall thickening of 2.7 cm over a length of 5 cm, adjacent to the splenic flexure, accompanied by fat impaction and turgid periparietal circulation (Figure 17).

The patient refused a lumbar puncture to assess brain involvement and establish the cause of brachial monoparesis, as well as a colonoscopy and transesophageal puncture of the cardiac tumor mass.

A treatment for relapsed/refractory T-cell acute lymphoblastic leukemia (T-ALL) with nelarabine/cyclophosphamide/etoposide (Nel-Cyclo-Etop) was initiated.

The initial course of treatment was favorable, characterized by the restoration of atrioventricular conduction and sinus rhythm within four days, as clinically observed and confirmed by dynamic EKG examinations. Additionally, the patient experienced resolution of brachial monoparesis. Consequently, the planned placement of a cardiac pacemaker was deemed unnecessary, as the patient emerged from complete heart block during chemotherapy and subsequently maintained a stable sinus rhythm.

Since 11 May 2023, the patient’s general condition deteriorated, with gait disturbances, myopathy, neurological degradation, and becoming dependent on oxygen. Although the heart rate stabilized at 100/min, dyspnea developed, along with oliguria, neutropenic colitis/toxic megacolon, pancytopenia, alterations in renal and hepatic tests, and electrolyte imbalances, leading to death due to multiple organ failure on 14 May 2023 (Table 2).

## 4. Discussion

Acute lymphoblastic leukemia (ALL) is a malignant disorder of lymphoid progenitor cells with an estimated annual incidence worldwide of approximately 1 in 100,000 and in Europe of around 4 cases per 100,000 (~5000 cases annually) [3,8]. The average 5-year overall survival rate for adult patients aged 18–60 years with acute lymphoblastic leukemia (ALL) ranges from 20% to 35%, despite high complete remission rates of 75–80% [5,10], though percentages may vary depending on the cited source [1,3].

The time to relapse is a strong predictor of long-term survival, with additional prognostic factors determined by the site of relapse, response to salvage therapy, stem cell transplant performance, and age [11].

Relapse predominantly occurs in the bone marrow, but also extramedullary (EMR), with a 5-year cumulative incidence of 41% and 5.8%, respectively [8]. The incidence of EMR relapse post-SCT in ALL is 12.9% vs. 4.6% in AML [12]. The 5-year survival rate after EMR is 18.5% in ALL and 0% in AML. For patients with acute lymphoblastic leukemia (ALL) who have undergone transplantation, the 3-year survival rate is significantly impacted by the site of relapse. Specifically, survival is 30.1% following extramedullary relapse (EMR) compared to just 8.5% for bone marrow relapse (BMR). [10]. Data on EMR in adults are limited, with most post-SCT relapse data referring to children [7]. In adults, late relapses at 5–10 years post-transplant are rarer, with our patient’s relapse occurring approximately 5 years after diagnosis and 4 years and 3 months after transplant. The main EMR relapse is in the CNS, followed by the testicles, followed by ocular relapse, with recurrences in other sites/sanctuaries being sporadically described in the literature, often isolated and generally followed by medullary relapse [13].

Recurrences have been reported in the breast, ovaries, uterus and cervix, pancreas and kidneys, intestines, heart, eyes, lymph nodes, and skin [14].

The risk factors for post-stem-cell transplantation (SCT) relapse include advanced disease at the time of transplant, hyperleukocytosis, pre-transplant extramedullary disease (EMD), the use of total body irradiation (TBI) for conditioning, unfavorable cytogenetics, and peripheral blood stem cell infusion—the latter being present in our patient. Approximately half of extramedullary relapses (EMRs) are associated with pre-transplant EMD. We evaluated whether the patient’s mediastinal tumor could have been a T-cell lymphoblastic lymphoma; however, given its 3-year evolution and the confirmed thymoma diagnosis by two reputable pathology laboratories, an aggressive lymphoma was ruled out. We also considered whether post-transplant mediastinal radiotherapy might have prevented subsequent relapses, although dynamic post-transplant imaging was negative [15]. Acute and chronic GVHD reduce the risk of BMR but do not protect against EMR [16]. Our patient experienced cutaneous graft-versus-host disease (GVHD), with a prompt response to methylprednisolone. It has been described in the literature that GVHD, particularly when involving the skin, does not have a significant impact on extramedullary relapses, which are often considered sanctuary sites for leukemia. This could reduce the graft-versus-leukemia (GVL) effect, potentially allowing leukemia to persist in extramedullary sites, despite a strong systemic immune response. Studies have suggested that GVHD may not sufficiently target these sanctuary sites, limiting its effectiveness in preventing extramedullary relapse. Patients who regain complete remission (CR) after their first extramedullary relapse (EMR) have a risk of secondary EMR comparable to patients who initially relapse only in the bone marrow after transplant [17].

In a study of 556 patients with acute leukemia (2000–2013), including 446 with AML and 112 with ALL, 258 recurrences were observed, out of which 31 had extramedullary recurrences (EMR). Among these, 19 out of 31 achieved complete remission (CR); moreover, out of these 19, 13 experienced another relapse (3 bone marrow relapses (BMR) and 10 EMR). Among the 10 patients with EMR, 8 achieved CR, and out of these 8, 6 relapsed again (1 BMR and 5 EMR) [18,19]. Although extramedullary relapses are reported in both subtypes of leukemia, they are not exclusive to acute lymphoblastic leukemia (ALL). It is likely that our patient follows this pattern of three secondary recurrences without medullary involvement.

The most common site of relapse is in the CNS, followed by the testicles [20,21].

In males, a rare but possible site of relapse is the testicles, with an incidence of 0.5–2% in children [21]. Testicular relapse has been reported in cases of complete leukemia remission in children. In adults, however, these cases are extremely rare, but they can represent the first sign of systemic recurrence [22,23,24]. The risk factors for testicular relapses include pubertal age, hyperleukocytosis, T-cell phenotype, and extramedullary involvement (such as CNS, mediastinal mass, and hepatosplenomegaly) [25]. The patient had ALL-T and hepatomegaly at the time of diagnosis. The testicles are considered an “immune sanctuary,” contributing to their predisposition for extramedullary relapse after hematopoietic stem cell transplantation (HSCT) [25]. Several mechanisms have been proposed to explain this, including immunological tolerance of the testicular microenvironment and specific factors favoring tumor cell growth and proliferation, like lymphocyte growth factors (IL like-1) [26]. The low temperatures in the scrotal sac compared to the rest of the body may decrease the cytotoxic effect of chemotherapy [12]. Pubertal physiological changes result in altered vascular endothelial permeability in the testicles and local immunosuppression, which could explain the lower number of testicular relapses in adults compared to children treated for ALL [27].

The properties of the blood–testis barrier, as well as the blood–brain barrier, are considered pharmacological sanctuaries, similar to other extramedullary sites, resulting in reduced chemotherapy penetrance at these levels, which has therapeutic implications [11,28]. Therefore, the appropriate choice of agents, such as methotrexate, which efficiently penetrate the blood–testis barrier, may play a role in preventing testicular relapse [29,30]. Some doctors have opted for chemotherapy alone in children, avoiding orchiectomy or testicular irradiation to preserve testicular function. Additionally, a longer maintenance therapy of 3 years is associated with fewer relapses, including testicular ones, but our patient did not require maintenance therapy after transplantation. Estrogen therapy has been shown to prevent Leydig cells from binding to leukemic cells and inhibit lymphocyte infiltration directly. However, the observations in this article suggest that further investigations are needed in order to explore this potential therapeutic approach in more depth [12]. Testicular relapse usually presents as a palpable mass, and both diagnostic and therapeutic roles are played by orchiectomy, followed by scrotal radiation therapy with 24 Gy or contralateral testicular biopsy allowing only prophylactic administration of 15 Gy. Because medullary or extramedullary relapse may follow EMR, most authors recommend consolidating the response with high-dose methotrexate chemotherapy. Successful management with orchiectomy alone has been reported, although it is not widely accepted [31]. New therapies, such as chimeric antigen receptor T-cell therapy, successfully treat testicular relapse with minimal side effects and could be further investigated [32]. Although we chose HyperCVAD therapy in this case, guided by the presence of abdominal lymphadenopathy, there is insufficient data regarding its ability to penetrate the blood–testis barrier, and, therefore, vigilant monitoring of these patients is imperative.

It is also important to differentiate testicular relapse in ALL from primary testicular tumors, such as testicular seminoma in elderly patients, as the imaging appearance may be similar. Other differential diagnoses should include testicular hematoma, testicular torsion (clinical distinction), epididymo-orchitis, and orchitis.

There are no long-term, large-scale studies available for adults with testicular relapse in ALL. Due to this fact, many case reports on adult ALL, including those related to extramedullary relapses, have often been compared to pediatric cases, which are more abundant since ALL is the most common acute leukemia and malignancy in children. However, among children, the 5-year overall survival rate has been reported to be approximately 73% [33]. The survival rate is lower for those with occult testicular relapse (approximately 53%) or early relapse [34]. This is because early testicular relapse may represent a high likelihood of systemic disease compared to late presentation, which could be managed locally [35].

As demonstrated in this case, the diagnosis and treatment of isolated testicular relapse in ALL can be very challenging, given the rarity of this entity and the lack of data and experience for the adult population. We did not find any descriptions in the literature of late testicular relapse in an adult with T-ALL allotransplanted, and one case (80-year-old patient) was observed in B-ALL Ph+ [36]. The case is unique, especially regarding the extended interval between initial treatment and relapse, which was supposed to be a favorable prognostic factor for the patient, with a reported 5-year survival rate in testicular EMR after 36 months of transplantation of 60% [12].

Additionally, the demonstration of relapse was performed using morphology and flow cytometry from seminal fluid. Immunophenotyping can theoretically be used on all fluids and processed tests; however, in our hematology department, it was only used for characterizing cells from peripheral blood, bone marrow, cerebrospinal fluid, and, in isolated cases, pleural fluid. In the literature, flow cytometry and molecular biology have been performed on aspirated fluid from the anterior chamber of the eye [24]. Since we had not tested flow cytometry on seminal fluid in our laboratory and found no description of its use for this purpose in the country or in the literature, performing this analysis was a challenge for the flow cytometry physician who developed the protocol adapted to the patient’s case. However, the definite morphological changes in massive relapse with blasts in seminal fluid prompted the immunophenotypic investigation, especially as it was a non-invasive method. The testicle measured 10 cm, was tense, hypervascularized, and would not have safely allowed testicular puncture. Intra-abdominal determinations would have been therapeutically approached late if orchiectomy per primam had been chosen with the risk of systemic relapse.

The third EMR relapse after CNS and testicular involvement is ocular relapse, cited in children with a frequency of 2.2% of relapsed ALL cases [34]. The frequency in adults is unknown, and sporadic reports exist [1,15,19]. The involvement includes the eye, orbit, or optic nerve. The retina is the most common site of ocular involvement in acute leukemias. Relapse is usually demonstrated by fine-needle aspiration biopsy. The eye represents a pharmacological sanctuary, and radiotherapy and chemotherapy using HD-MTX represent the therapeutic solution. A second transplant may increase survival rates. CAR T therapy in relapsed B-ALL has not proven effective, although it efficiently controls the disease in medullary and CNS relapses [37,38]. Due to the immunosuppression caused by the disease and treatments used, viral and bacterial ocular infections, neuritis, uveitis, pseudohypopyon, hemorrhage, vascular occlusion, and retinal detachment often occur [39]. Usually, disease relapse is associated with CNS involvement (75%) or medullary (55%), and isolated ocular relapse is rare [18,20]. Thus, lumbar puncture is mandatory for all patients with ocular relapse. Ocular involvement alters prognosis, halving 5-year survival compared to those without ocular involvement. Cases of ocular relapse have been described in both T-ALL and B-ALL. Typically, orbital extramedullary determinations occur at diagnosis and more rarely during the disease course. In 2021, seven cases of orbital determination at diagnosis and one case preceding the diagnosis of ALL were described [1]. However, only two descriptions of post-transplant orbital relapse were found, none of T-ALL, none at 5 years post-transplant, and none after a previous testicular relapse [40,41]. The fact that the patient did not have concomitant cerebral involvement with ocular involvement demonstrates the importance of radiotherapy during induction.

In acute leukemia, cardiac involvement postmortem has been demonstrated in 30–40% of patients, usually in the late stages of the disease, more frequently in AML than in ALL, with most being asymptomatic [39]. The frequency of antemortem cardiac involvement is unknown and difficult to demonstrate [21,37]. The most common cardiac involvement has been described in children, usually accompanied by marrow involvement and blast discharge into the blood. An isolated cardiac EMR is followed by medullary relapse in 5 months. The diagnostic methods used include CT, TTE, CMR, and PETCT. The certainty diagnosis is made by biopsy from the cardiac mass, which is difficult and risky to approach, or pericardial biopsy.

In a study from 1985 to 2021, 30 cases of cardiac involvement at diagnosis were detected. The presentation modalities included eight cases of cardiac tamponade, all ALL-T; nine cases of cardiac mass, eight B-ALL and one T-ALL (right ventricular involvement was found more frequently than left ventricular involvement); eight cases of myocardial hypertrophy, five LV, two IV septa, one IA septum; six T-ALL phenotypes; and six cases of myocardial infarction [39]. The management of patients with cardiac leukemic infiltration is challenging due to the cardiotoxic medications and previous radiotherapy used. Cardiotoxicity from previous treatments is challenging to assess, and that from subsequent treatments is even more so. The size and location of the tumor mass define the symptoms. Moreover, an LV apex hypermobile mass could cause outflow tract obstruction and thus reduce cardiac output, leading to insufficient coronary perfusion and angina. The LV-free wall infiltration could cause a reduced left ventricular ejection fraction (LVEF). Patients with severe hemodynamic disturbances may require cardiovascular surgery. Five patients experienced resolution of the cardiac mass under chemotherapy between 5 days and 2 months after chemotherapy [22,42].

CAR-T therapy and donor lymphocyte infusion manage extramedullary relapse, but their role in cardiac extramedullary relapse is unclear and has a high toxicity profile [22,43].

We have found in the literature eight cases of relapsed post-transplant acute leukemia at the cardiac level. Among them, two were diagnosed with T-ALL and the rest with B-ALL [44].

The management of cardiac damage is difficult and must aim at the complications that appear, mainly atrio-ventricular conduction disorders or ventricular rhythm disorders and heart failure phenomena that occur as a result of infiltration of the heart muscle [45]. The response to chemotherapy treatment can be favorable. The biggest challenge occurs when the patient first addresses the cardiologist, because establishing the etiology of cardiac manifestations, if it can be established, can take a long time [45,46].

Isolated colonic relapse is rare. The persistence of diarrhea syndrome in the absence of infections and post-transplant intestinal GVHD requires colonoscopy with biopsy. We found three relapse cases after alloSCT, but which also associates medullary relapse [47,48,49] (Table 3).

## 5. Conclusions

For patients with acute lymphoblastic leukemia (ALL) who undergo transplantation, the majority of extramedullary relapses (EMR) occur within the first two years post-transplantation [50]. In adults, post-transplant extramedullary relapses are rare and typically reported as case presentations in the literature, often accompanied by subsequent marrow relapse, and are frequently refractory to treatment [51]. Isolated extramedullary relapses can be followed by additional extramedullary occurrences [52]. The diagnosis of relapse is established through aspiration, biopsy, or ablation of the affected tissue or organ [36].

Notably, our literature review did not identify any documented cases of testicular relapse confirmed by morphology and immunophenotyping of spermatic fluid. Additionally, we found no reports of leukemic involvement in the testicle, orbit, heart, or colon. Specifically, the occurrence of leukemia in these four organs—testicle, orbit, heart, and colon—in adults with T-cell ALL and relapses occurring distant from transplantation has not been documented.

## Figures and Tables

**Table 1:** Laboratory tests necessary for the diagnostics of ALL pro-T in our patient.

Laboratory Analyses	Results
HLG	Lc = 4930/mm^3^, 0% neutrophils, 96% lymphocytes cu 50% lymphoblasts. Hb = 12.9 g/dL, Pl = 81,000/mm^3^
Medullary aspirate	Lymphoblast cells ~56%: Some small cells, with scant cytoplasm, hyperbasophilic-ALL
Cytochemistry	PAS (Periodic Acid–Schiff) reaction: 20% positive elements (large, multiple granules). Peroxidase and alpha naphtyl acetate esterase (ANAES) reactions negative
Bone marrow immunophenotyping	Population percentage of 30% expressing: CD45+ weak, icCD3+, sCD3−, CD7+, CD99+ weak, CD34−, CD1a−, CD4−, CD8−, CD2−, CD5+ weak, CD123+ weak, MPO−, CD33− Erythroblasts 30%, mature lymphocytes 35%, the vast majority being T lymphocytes [9], The phenotypic profile was suggestive of the diagnosis of acute lymphoblastic leukemia T (pro-T ALL)
Bone marrow Histopatological and imunohistochimical	Diffuse marrow infiltration within a lymphoproliferative process with lymphocytes: CD3, CD5—positive in atypical T lymphocytes (90% of the marrow cellular population) CD20—positive in B lymphocytes in a few small lymphoid follicles CD10—negative BCL2—positive in the atypical lymphoid population Ki67 30%
Karyotype	46 XY
Molecular Biology	No changes. Negative for: TCF3::PBX1-t(1;19)(q23;p13); KMT2A::AFF1-t(4;11)(q21;q23) BCR::ABL1 p190-t(9;22)(q34;q11); BCR::ABL1 p210-t(9;22)(q34;q11) ETV6::RUNX1-t(12;21)(p13;q22); SIL-TAL 1-del(1)(p32;p32)
HLA	Compatible 100% HLA with his sister

**Table 2:** Medical events and therapeutic interventions from diagnosis to extramedullary relapses (first, second, and third) and ultimately to death.

Date	Medical Events	Therapeutic Behavior and Result
2013	Mediastinal Tumor	Neglected
November 2016	Thymoma 13/14/15 cm	Thymomectomy—December 2016
June 2017	Pro-T ALL	induction and consolidation gmall, 2003 cerebral radiotherapy, 24 gy intrathecal (mtx, ara-c, dexamethasone) result: complete remission
February 2018	allotransplant sister	immunosuppression, 6 months (methotrexate and tacrolimus, then sirolimus, followed by methylprednisolon and sirolimus)
March 2022	SARS-CoV-2	home treatment
June 2022	first extramedullary relapse left testicular—diagnostic immunophenotyping of semen	HyperCVAD Block A radiotherapy, 24 gy/12 sessions for the scrotal sac and the lymph nodes along the left spermatic vein result: complete metabolic remission petct
January 2023	second extramedullary relapse bilateral ocular left eye clinically, imaging, and biopsy demonstrated right eye demonstrated by imaging	methotrexate, asparaginase, calcium folinate radiotherapy, 30 gy/15 sessions result: imaging complete remission
2 May 2023	third emr: cardiac and colonic mass (+cns?)	nelarabine+cyclophosphamide+ etoposide
14 May 2023	death	msof

**Table 3:** The review of cases in the literature on extramedullary relapses in cardiac, colonic, and orbital locations after allo-HSCT for ALL, and the unique case of testicular relapse in an adult following allo-HSCT.

Cardiac EMR After Allo-HSCT Citation	Age, Years Old (y.o.)	Description of Cardiac Involvement/Injury and Manifestation	
2002 Wright, T. et al. [44]	33 y.o. man	2 years after bone marrow transplant from a matched unrelated donor: isolate intracardiac mass	B-ALL t (11;19)
2003 B C A M Bekkers et al. [42]	44 y.o.man	After allotransplant: atrial fibrillation, ventricular tachycardia, complete atrioventricular block with an escape rhythm originating from the posterior fascicle, died from ventricular fibrillation the following day At necropsy, extensive cardiac localisation and concomitent relapse of bone marrow	B-ALL
2006 Tsukasa, H. et al. [43]	14 y.o. Female	2 stem cell transplantation: 1: using bone marrow; 2: using peripheral blood Isolated cardiac relapse was diagnosed using several non-invasive imaging techniques Developed progressive and fatal hematological disease	T-ALL
2016 Kiju Chang et al. [45]	42 y.o. man	7 months after allo-HSCT from a matched sibling donor Surgical LV biopsy revealed diffuse infiltration of ALL blasts in the myocardium and pericardium, complete atrioventricular block, heart failure, multiple heart attacks	B-ALL
2017 Baritussio [46,47]	38 y.o. man	9 months after allotransplantation by infiltration in the eyes, myocardium, and pericardium Flocytometric MO and CSF 0.09% T-lymphoblastic lymphocytes were highlighted. He was treated with nelarabine PEV and intrathecal cytosar, with the resolution of the eye and heart tumor	T-ALL
2018 Nadel, J [48]	38 y.o.	Endomyocardial relapse and PB relapse	B-ALL Ph positive
2021 Sheikh, I. et al. [22]	11 y.o. man	HSCT with a single unrelated umbilical cord blood unit 8 months post-transplant developed cardiorespiratory failure, BAV, leukemic infiltration of SIV and LV, which required the implantation of a cardiac pacemaker. Later, a cardiac biopsy was performed and chemotherapy was initiated	B-ALL
2023 Cao., Yigeng et al. [49]	42 y.o. female	Allo-HSCT from a matched, sibling donor CART-cell therapy A cardiac mass was revealed after 11 months allo-HSCT, 5 months after CART	B-ALL/LBL
**Colon EMR after allo-HSCT** **citation**	**Age,** **years old** **(y.o.)**	**Description of colonic involvement/injury**	
2019 Hathorn, K [50]	57 y.o. man	27 months from transplant relapse PB and BM transverse colon infiltrate	(Ph+) ALL
Issak, A [51]	61 y.o.	Sigmoid infiltrated and BM infiltrated	B-ALL
2022 Ifthikar, Zainab [52]	30 y.o. male	Allo-HSCT from matched sibling Second allo-HSCT from a different matched sibling donor Acute lower GI bleeding Extramedullary relapse of ALL in the ascending colon+ relapse BM and PB	B-ALL
**Orbital EMR after allo-HSCT** **citation**	**Age,** **years old** **(y.o.)**	**Description of orbital involvement/injury**	
1995 A, Colombini [41]	13 y.o. girl	3 years after BMT A tumor at the inferomedial part of the orbit infiltrating the maxillary and ethmoid sinuses and nasal cavities and also involving the rectus muscles	B-ALL
2020 Willier, S [38]	3 y.o. boy	Two allogeneic hematopoietic stem cell transplantations (HSCT) and previous CD19-CAR T-cell therapy. On day +16 after second transplant, episcleral and intraocular infiltration ALL relapse, and after few days, PB and BM relapse	pro-B ALL
**TESTIS-adult EMR after allo-HSCT** **citation**	**Age,** **years old** **(y.o.)**	**Descrition of testis involvement/injury**	
2019 Jayakrishnan, T. [36]	80 y.o. man	5 years post-allogeneic matched unrelated donor Peripheral blood stem cell transplant	B-ALL Ph+

## Data Availability

The original contributions presented in the case report are included in the article, further inquiries can be directed to the corresponding authors.
